# Prophylaxis for patients at Risk to Eliminate Post-operative Atrial Fibrillation (PREP-AF trial): a protocol for a feasibility randomized controlled study

**DOI:** 10.1186/s13063-021-05318-1

**Published:** 2021-06-07

**Authors:** Heather A. Smith, Salmaan Kanji, Diem T. T. Tran, Calum Redpath, Dean Ferguson, Tori Lenet, Greg Sigler, Sebastien Gilbert, Donna Maziak, Patrick Villeneuve, Sudhir Sundaresan, Andrew J. E. Seely

**Affiliations:** 1 Division of General Surgery, Department of Surgery, University of Ottawa, Ottawa, Canada; 2 Department of Pharmacy, The Ottawa Hospital and Ottawa Hospital Research Institute, Ottawa, Canada; 3 Division of Cardiac Anesthesiology, Department of Anesthesiology & Pain Medicine, University of Ottawa Heart Institute, Ottawa, Canada; 4 University of Ottawa Heart Institute, Ottawa, Canada; 5 Ottawa Hospital Research Institute, Ottawa, Canada; 6 Division of Thoracic Surgery, Department of Surgery, University of Ottawa, Ottawa, Canada; 7 Division of Thoracic Surgery, Department of Surgery and Critical Care Medicine, University of Ottawa, Ottawa, Canada

**Keywords:** Postoperative atrial fibrillation, Prophylaxis, Prevention, Arrhythmia, Thoracic surgery

## Abstract

**Background:**

Postoperative atrial fibrillation (POAF) is a frequent adverse event after thoracic surgery with associated morbidity, mortality, and healthcare costs. It has been shown to be preventable with prophylactic amiodarone, which is only recommended in high-risk individuals due to the potential associated side effects. Risk factors for POAF have been identified and incorporated into a prediction model to identify high-risk patients. Further evaluation in the form of a multicenter clinical trial is required to assess the effectiveness of prophylaxis specifically in this high-risk population. The feasibility of such a trial first needs to be assessed.

**Methods:**

The PREP-AF trial is a double-blind randomized controlled feasibility trial. Individuals undergoing major thoracic surgery who are identified to be high-risk by the POAF prediction model will be randomized 1:1 to receive a short course of amiodarone vs. placebo in the immediate postoperative period. The primary outcome is feasibility, which will be measured by the number of eligible patients identified, consented, and randomized; intervention adherence; and measurement of future outcomes of a full trial.

**Discussion:**

This study will determine the feasibility of a randomized controlled trial to assess the effectiveness of prophylactic amiodarone, in high-risk patients undergoing major thoracic surgery. This will inform the development of a multi-center trial to establish if prophylactic amiodarone is safe and effective at reducing the incidence of POAF. Preventing this adverse event will not only improve outcomes for patients but also reduce the associated health resource utilization and costs.

**Trial registration:**

ClinicalTrials.gov NCT04392921. Registered on 19 May 2020.

## Administrative information

The order of the items has been modified to group similar items (see http://www.equator-network.org/reporting-guidelines/spirit-2727-statement-defining-standard-protocol-items-for-clinical-trials/).

**Table:** 

**Title {1}**	Prophylaxis for patients at Risk to Eliminate Post-operative Atrial Fibrillation (PREP-AF trial): a protocol for a feasibility randomized controlled study
**Trial registration {2a and 2b}.**	Registry: clinical trials.gov (NCT04392921) Date of Registration: May 19, 2020
**Protocol version {3}**	May 19, 2020
**Funding {4}**	TOHAMO Innovation Fund Grant The funding body had no role in the design of the study; collection, analysis, and interpretation of data; and in writing the manuscript.
**Author details {5a}**	*^†^Heather Smith, MD, MSc, Division of General Surgery, Dep’t of Surgery, University of Ottawa hsmit037@uottawa.ca ^†^Salmaan Kanji, Pharm.D, Department of Pharmacy, The Ottawa Hospital and Ottawa Hospital Research Institute, skanji@toh.ca Diem T. T. Tran MD, MSc, FRCPC, Division of Cardiac Anesthesiology, Dep’t of Anesthesiology & Pain Medicine, University of Ottawa Heart Institute, dtran@ottawaheart.ca Calum Redpath, MD, MSc, FRCSC, University of Ottawa Heart Institute, credpath@ottawaheart.ca Dean Ferguson, PhD, Ottawa Hospital Research Institute, dferguson@ohri.ca Tori Lenet, MD, Division of General Surgery, Dep’t of Surgery, University of Ottawa, tlenet@toh.ca Greg Sigler, MD, Resident, Division of General Surgery, Dep’t of Surgery, University of Ottawa, gsigler@toh.ca Sebastien Gilbert MD, FRCSC, Division of Thoracic Surgery, Dep’t of Surgery, University of Ottawa, sgilbert@toh.ca Donna Maziak, MDCM, MSc, FRCSC, Division of Thoracic Surgery, Dep’t of Surgery, University of Ottawa, dmaziak@toh.ca Patrick Villeneuve, MDCM, PhD, FRCSC, Division of Thoracic Surgery, Dep’t of Surgery, University of Ottawa, pjvilleneneuve@toh.ca Sudhir Sundaresan MD, FRCSC, Division of Thoracic Surgery, Dep’t of Surgery, University of Ottawa, ssundaresan@toh.ca^†^Andrew J.E. Seely MD, PhD, FRCSC, Division of Thoracic Surgery, Dep’ts of Critical Care Medicine and of Surgery, University of Ottawa *Corresponding Author; ^†^Principal Investigator
**Name and contact information for the trial sponsor {5b}**	Andrew J.E. Seely MD, PhD, FRCSC Thoracic Surgeon and Critical Care Medicine Specialist Professor, University of Ottawa Scientist, Ottawa Hospital Research Institute Vice-Chair Research, Dep’t of Surgery, University of Ottawa Director of Research, Division of Thoracic Surgery e-mail: aseely@toh.ca Telephone: (613) 737-8899, ext. 74052 Fax : 613-737-8668
**Role of sponsor {5c}**	AS led the study design; will lead collection, management, analysis, and interpretation of data; reviewing of the report; and the decision to submit the report for publication.

## Introduction

### Background and rationale {6a}

Postoperative atrial fibrillation (POAF) is the most commonly sustained arrhythmia after thoracic non-cardiac surgery [1, 2]. We found it occurred in 17% of patients after lobectomy, 23% after pneumonectomy, and 12% after esophagectomy, making it the most common postoperative adverse event after thoracic surgery at our institution [3]. It is associated with increased postoperative morbidity, length of stay, ICU admission, and mortality [1, 2]. For example, POAF is associated with increased mortality risk after esophagectomy (from 4.8 to 8.1%, *p* = 0.04) [4]. Recently, the results of two large multicenter randomized controlled trials (RCTs) (POISE-1: metoprolol vs. placebo and POISE-2: aspirin vs. placebo) were analyzed with regard to the impact of POAF. Among 18,117 patients (mean age 69 years, 57.4% male), 404 had POAF (2.2%). The stroke incidence 1 year after surgery was 5.58 vs. 1.54 per 100 patient-years in patients with and without POAF. Patients with POAF also had an increased risk of death (incidence 31.37 vs. 9.34; aHR 2.51, 95% CI 2.01–3.14; *P* < 0.001) and MI (incidence 26.20 vs. 8.23; aHR 5.10, 95% CI 3.91–6.64; *P* < 0.001) [5]. Given the frequent incidence after thoracic surgery and marked impact of POAF, prophylaxis to prevent POAF is of interest.

#### POAF prophylaxis and safety

Of the agents that have been used for prophylaxis, metoprolol and amiodarone are most effective at preventing POAF [2, 6]. Unfortunately, the use of metoprolol for prophylaxis in a generalized manner after non-cardiac surgery is associated with increased risk of stroke and death [7]. Amiodarone, a Class III anti-arrhythmic, has been extensively studied as a prophylactic agent for POAF, effectively reducing the rate of POAF by as much as 31% after thoracic surgery, with a total relative risk (RR) of 0.22 (*P* = 0.001; 95% CI, 0.09–0.54) and a NNT of 5.1 [6]. However, the safety of prophylactic amiodarone has also been controversial; high dosing regimens have been associated with hypotension, bradycardia, QT prolongation, and in patients undergoing pneumonectomy, adult respiratory distress syndrome (ARDS), in addition to more common effects of tremor, nausea, and constipation [8]. Several recent trials have suggested administration of low dose prophylactic amiodarone to patients undergoing non-cardiac thoracic surgery to be safe [9–13]. Given its effectiveness, but concern regarding toxicity, the Society of Thoracic Surgeons (STS) most recent 2011 guidelines only recommend amiodarone prophylaxis for patients undergoing esophagectomy, and pulmonary resection, excluding pneumonectomy, until additional data addressing its potential for pulmonary toxicity is available (class III recommendation) [14]. The American Association of Thoracic Surgeons 2014 guidelines recommends surgeons consider prophylactic amiodarone for the prevention of POAF for intermediate and high-risk patients undergoing pulmonary resection (class IIa recommendation) and for all patients undergoing esophagectomy (class IIb recommendation) to minimize potential side effects of amiodarone [1]. However, until recently, there have been no validated tools to identify patients at elevated risk of POAF.

#### Prediction of POAF

In reviewing the literature for risk stratification tools to predict POAF after thoracic surgery, we only identified one which was internally validated for all patients undergoing major thoracic surgery and contained factors obtainable in the preoperative period (resting heart rate, gender, and age) [4, 15, 16]. After evaluation of this tool at our institution in a sample of 2036 patients undergoing major thoracic surgery between 2008 and 2018, we found it to also be externally valid [17]. To facilitate identifying potential candidates for prophylaxis, we translated this POAF risk model into a designation of high vs low risk. Taking into account the cited importance of the extent of surgery in POAF incidence, we designated the “high risk” patients as individuals undergoing a procedure with extensive pericardial dissection (esophagectomy, pneumonectomy, and lobectomy) with a risk score of 4–6/6. This high risk group represented 51% all patients undergoing major thoracic surgery and experienced a fourfold higher incidence of POAF compared to the remaining “low risk” cohort (12.3% vs 3.4%, *p* < 0.001). If amiodarone provides a risk ratio of 0.22 (as demonstrated by the meta-analysis by Zhang et al.) in the “high-risk group”, it would reduce the POAF rate to 2.7% which would translate to a NNT of 10.4 [6].

While there is potential benefit of amiodarone in reducing POAF, there are risks with its administration and further research is needed to understand its effectiveness and safety in patients with increased risk of POAF as highlighted by the most recent guidelines [14]. An experimental randomized design is necessary to remove important biases and to augment the internal validity of the study to the point that it will be given adequate credence and effect on clinical practice. Our overall aim is to decrease the incidence of POAF after thoracic surgery by specifically targeting high-risk patients with best evidence prophylactic therapy.

### Objectives {7}

This study aims to assess the feasibility of a blinded, randomized controlled trial where patients at increased risk of POAF who are undergoing major thoracic surgery are randomized to receive prophylactic amiodarone or placebo. This is intended to inform the development of a multi-center randomized controlled trial to establish if prophylactic amiodarone is safe and effective at reducing the incidence of POAF after major thoracic surgery.

### Trial design {8}

The PREP-AF feasibility trial is a pragmatic single-center, parallel group, double-blinded, randomized controlled feasibility study.

Individuals will first be assessed for their risk of POAF based on their POAF prediction score (Table 1) and planned procedure.

**Table 1:** POAF prediction score [15, 17]

Criteria	Points
Male gender	1
HR^a^ >72 bpm	1
Age 55–74 years	3
Age >75 years	4
Total	/6

^a^Heart rate (HR)

Individuals will be considered “high-risk” of POAF by meeting both of the following criteria: (1) POAF prediction score greater than or equal to 4 (Table 1) and (2) undergoing lobectomy, extended lobectomy, pneumonectomy, or esophagectomy.

High-risk individuals will be randomized 1:1 to intervention or control group.

Individuals will be considered “low-risk” of POAF by one the following criteria: (1) POAF prediction score less than 4 (Table 1) or (2) undergoing gastrectomy or sublobar resection.

Low-risk individuals will receive standard care with monitoring for clinical outcomes and adverse events.

Patients using beta-blockers at the time of enrollment will be eligible to participate in the trial. Given that beta-blocker use is protective against development of POAF, we will stratify these patients at the time of randomization to ensure the proportion of beta blocker usage is equal between intervention groups.

## Methods: participants, interventions, and outcomes

### Study setting {9}

This study will be conducted with the Division of Thoracic Surgery at The Ottawa Hospital, an academic teaching center in Ottawa, Ontario, Canada. At our center, approximately 200 patients undergo major thoracic surgery annually. Of which, 52% undergo a lobectomy, 25% a sublobar resection, 5% a pneumonectomy, and 11% an esophagectomy.

### Eligibility criteria {10}

All patients undergoing thoracic surgery will be screened for eligibility. In order to participate, patients must provide consent if “high-risk” of POAF, be ≥18 years, and undergoing major non-cardiac pulmonary or esophageal surgery at the Ottawa Hospital during the 1-year time period.

Patients will be excluded if they are <18 years; are pregnant; have a history of atrial arrhythmia, Wolf-Parkinson-White syndrome (WPW), or 2nd or 3rd degree heart bock without a pacemaker; are currently on antiarrhythmic therapy; have a previous severe adverse reactions or contraindications to amiodarone; have QTc interval longer than 450ms; or have serum alanine transaminase or aspartate transaminase over 3 times the upper limit of normal, or Child-Pugh class C liver dysfunction.

Patients enrolled in other clinical trials are potential candidates for PREP-AF feasibility.

### Who will take informed consent? {26a}

All eligible individuals will be approached for participation in the trial by a research coordinator during their preoperative visit to discuss the study protocol, review eligibility criteria, and assess willingness to participate. If high risk, a consent discussion will be initiated, including a discussion of the risk and benefits of participating in the trial.

### Additional consent provisions for collection and use of participant data and biological specimens {26b}

Not applicable.

### Interventions

#### Explanation for the choice of comparators {6b}

Placebo was selected as a comparator because no prophylactic agent is given as part of standard of care at our institution. In our survey of thoracic surgeons in Canada, only 5 of 67 respondents (7.4%) consider prophylaxis routinely.

#### Intervention description {11a}

Eligible high-risk patients will be randomized 1:1 to two treatment arms: *Intervention* patients will undergo the following: (a) postoperative day (POD) 0: receive 1050mg of amiodarone in 500mL of 5% dextrose in water (D5W) administered intravenously (IV) initiated at the time of anesthesia induction over 24 h at a rate of 0.73mg/min (43.75ml/h); (b) POD 1 to 5 or day of discharge (whichever occurs first):

- *◦ If able to tolerate po intake:* 400mg po BID for postoperative days 1 to 5 or until the day of discharge (whichever occurs first).
- *◦ If unable to tolerate po intake:* 1050mg IV in 500mL of 5% dextrose administered over 24h for postoperative days 1 to 5 or until the day of discharge (whichever occurs first).

*Control* patients will receive identically marked IV infusions or oral capsules, containing D5W or placebo. These dosing regimens, in keeping with published protocols, are considered safe in thoracic surgery patients [9, 11].

#### Criteria for discontinuing or modifying allocated interventions {11b}

Based on the known potential risks associated with short-term amiodarone administration, the following are indications of discontinuation: allergic reaction; hypotension (systolic BP<90); bradycardia (heart rate <50bpm); AV blockade (PR interval prolonged on electrocardiogram >0.20 seconds); ARDS (i.e., if unexplained progressive hypoxemia (berlin definition) with PF ratio <200); and QT prolongation (i.e., heart rate-corrected QT interval prolonged to >500ms) [18–22].

Given that amiodarone is also a treatment option for POAF, we will reveal the allocation group of randomized patients who develop POAF (defined as an irregularly irregular atrial rhythm without clear P waves, recorded on a standard 12-lead electrocardiogram (ECG) that occurred in the immediate 30-day postoperative period [3]) to allow clinicians to provide appropriate treatment doses of amiodarone.

#### Strategies to improve adherence to interventions {11c}

No additional strategies will take place to improve adherence to this trial, in keeping with the pragmatic design of this study.

#### Relevant concomitant care permitted or prohibited during the trial {11d}

Both groups will receive standard postoperative care in accordance to the existing pathways for postoperative thoracic surgical care at our institution. This includes routine postoperative electrolyte monitoring and replacement (during the inpatient stay for the first 3 days postoperatively). All patients will receive routine continuous heart rate monitoring for 48–72 h after surgery, discontinued at clinical discretion. Management of POAF will be guided by the published algorithm developed at our institution with the final decisions regarding rate control therapy and anticoagulation left to the discretion of the treatment team and with involvement of the cardiology team where appropriate [23].

#### Provisions for post-trial care {30}

No direct compensation will be allocated to participants.

### Outcomes {12}

The primary objective of this study is to demonstrate feasibility, inclusive of enrollment, randomization, intervention allocation, and monitoring of a randomized controlled trial. This will include the following outcome measures:

1. *Capability of enrollment* will be determined by an average of ≥1 patients enrolled per week.
  - We will also assess following outcomes: proportion of patients risk stratified and screened, proportion of eligible individuals consenting to involvement in this study, and proportion of recruited individuals who are enrolled in the study.
2. *Feasibility of the randomization processes* will be evaluated determined by ≥90% of enrolled high-risk patients randomized to an intervention or control group.
3. *Feasibility of blinding* of participant, care provider, investigator, and outcomes assessor to the intervention allocation of participants will be evaluated by ≥80% of respondents not aware of allocation as indicated in a survey to assess their knowledge of which patients received the intervention and placebo.
4. *Intervention delivery* will be assessed by determining if protocol adherence rates exceed ≥90% and recording observational data on the quality of intervention delivery using a data collection sheet.
5. *Monitoring of protocol deviation and compliance* measured by the frequency, rate, and rationale of events when study activities diverge from the REB-approved protocol.
6. *Monitoring of safety* will be assessed by determining the rate and efficiency of reporting adverse events if they occur and monitoring adherence rates to safety and monitoring protocols.
7. *Feasibility of data extraction analysis* will be determined if the required data could be abstracted for ≥90% of patients: medication use, incidence of postoperative atrial fibrillation, postoperative outcomes, etc.
8. *Resources* required to conduct a future multicenter PREP-AF trial will be assessed by evaluating the administrative capacity of the POAF research team, including the required number of hours of research assistant time, as well as the feasibility of the designated study budget.

Secondary clinical outcomes will include the incidence and severity of POAF (defined as an irregularly irregular atrial rhythm without clear P waves, recorded on a standard 12-lead electrocardiogram (ECG) that occurred in the immediate 30-day postoperative period [3]), and classified in severity by the Ottawa Thoracic Morbidity and Mortality (TM&M) system [3]; hospital length of stay; and incidence of other postoperative complications using the taxonomy of the TM&M definitions [3].

### Participant timeline {13}

Below is a schedule of activities that would be involved in trial participation:

**Table:** 

	Study period							
	Enrolment	Allocation	Post-allocation					Close-out
	Screening visit	Screening visit	Preoperative anesthesia visit		Operative hospital admission		30-day follow-up	
Timepoint**	***-t***_***1***_	0	***t***_***1***_	***t***_***2***_	***t***_***3***_	***t***_***4***_	***etc.***	***t***_***x***_
**Enrolment:**								
**Eligibility screen**	X							
**Informed consent**	X							
**Allocation**		X						
**Interventions:**								
***Amiodarone***					X			
***Control***					X			
***Standard of care***					X			
**Assessments:**								
Medical history, medication, demographics	X	X						
Height and weight, ECG			X	X				
Postoperative outcomes; adverse event review and evaluation					X	X	X	X
Complete case report forms	X	X	X	X	X	X	X	X

#### Sample size {14}

No sample size calculation was conducted for this feasibility study. We aim to recruit 80 patients for randomization during this 12-month trial. Our institution conducts approximately 200 major thoracic surgeries annually and we anticipate 100 of those patients to be eligible for inclusion based on POAF risk and assume a recruitment rate of 80. Patients will follow the schedule activities described in the “Participant timeline {13}” section including a 30-day follow-up.

#### Recruitment {15}

Eligibility criteria will first be assessed by the thoracic surgery physician team (surgeon, fellow, and/or resident) and the research coordinator at the time of the preoperative thoracic surgery clinic visit. All eligible individuals will be approached for participation in the trial initially by their thoracic surgery physician team after consenting to major thoracic surgery, and if interested, then approached by a research coordinator immediately after their thoracic surgery clinic visit. During this screening visit, the research coordinator will discuss the study protocol, review eligibility criteria, and assess willingness to participate. If the individual is “high-risk”, a consent discussion will be initiated, including a discussion of the risk and benefits of participating in the trial as outlined in the consent form. Their questions will be answered, and if interested and willing, then written informed consent will be obtained and the research coordinator will enroll the patient in the study.

If “low-risk”, no formal consent discussion will be conducted and the patient will continue with standard of care.

### Assignment of interventions: allocation

#### Sequence generation {16a}

After written informed consent is obtained, high-risk patients will be randomly assigned to either the intervention or control group by the clinical trial Pharmacy Research Technician using a secure web-based randomization system at the Methods Centre of The Ottawa Hospital Research Institute. Randomization will be stratified according to pre-randomization beta-blocker use. Allocation bias will be prevented by using a randomization table with variable blocks of 4 and 6.

#### Concealment mechanism {16b}

Patients, physicians, nurses, and the research coordinator will be kept strictly blinded as to individual group allocation and treatment. The intervention, active or placebo, will be provided to the clinicians responsible for administration in concealed containers. The clinical trial Pharmacy Research Technician will be responsible for the coding and labeling of all containers and instructions for administering the intervention will be identical for both control and amiodarone groups.

#### Implementation {16c}

The research coordinator will enroll recruited patients in the trial. The clinical trial Pharmacy Research Technician will assign enrolled individuals to an intervention using a secure web-based randomization system in accordance to the allocation sequence provided by the Methods Centre of The Ottawa Hospital Research Institute. Only the Pharmacy Research Technician at the Ottawa Hospital will see the randomization table and treatment assignments.

### Assignment of interventions: blinding

#### Who will be blinded {17a}

All members of the surgical care team including both in the operating room and on the surgery unit will be blinded to the intervention. The Pharmacy Research Technician preparing the medication will not be blinded to ensure appropriate allocation.

#### Procedure for unblinding if needed {17b}

If unblinding is required, physicians may login to the trial website to reveal the allocated treatment arm of the patient. The website will be available 24/7 for clinicians. It will also be accessible to the investigators to track if and when unblinding of participants occurs.

### Data collection and management

#### Plans for assessment and collection of outcomes {18a}

All data will be collected by trained research assistants. Data will be entered by the research assistants on password-protected files stored on the institutional network from the following: the electronic and paper case report forms developed and maintained specifically for this study; data from the Thoracics Morbidity & Mortality database (a clinical database from prospectively populated by the thoracic surgical team that is used to record complications of patients undergoing thoracic surgery at our institution) [3]; and The Ottawa Hospital Electronic Medical Records which will provide duplicate measurements to enhance data quality. Patient randomization and allocation data will be entered by the Research Pharmacy Technician.

#### .Plans to promote participant retention and complete follow-up {18b}

No additional strategies will take place to improve retention and completion by participants in this trial, in keeping with the pragmatic design of this study.

#### Data management {19}

Study data will be kept in password-protected files and stored on the institutional network with access restricted to the research team. A different user (i.e., one who has not performed the original data entry) will randomly select 20% of the collected CRF forms to review and verify for accuracy. Data will be coded by the research team for analysis. A Data Management Plan (DMP) developed in collaboration with the Methods Centre of the Ottawa Hospital Research Institute provides further details regarding data management procedures.

An independent Data Safety and Monitoring Board (DSMB) consisting of one pharmacist and three clinician scientists will perform data safety monitoring. The DSMB will be responsible for assuring that study participants in PREP-AF are not exposed to unnecessary or unreasonable risks and that the study is being conducted according to the highest scientific and ethical standards. Conduct of the DSMB is outlined in the PREP-AF DSMB Terms of Reference.

#### Confidentiality {27}

Patient’s personal health information will be kept confidential, unless release is required by law or permission is given by the patient. The consent form makes note that representatives of the local Research Ethics Board (REB) and/or Health Canada may review original medical records. A master list of the patient identifiers will be stored in a separate password protected, encrypted file on a secure institutional network at the Ottawa Hospital with restricted access to those involved as per the study protocol. All other electronic records will contain de-identified and coded data which will be password protected and encrypted.

#### Plans for collection, laboratory evaluation, and storage of biological specimens for genetic or molecular analysis in this trial/future use {33}

Not applicable to the nature of this study.

### Statistical methods

#### Statistical methods for primary and secondary outcomes {20a}

The primary end point of this study is feasibility so no in-depth statistical analysis is planned. However, for the anticipated full-scale trial to follow, we plan to conduct analysis using an intention-to-treat, considering all patients as randomized regardless of whether they received the randomized treatment. Patients will be administered the intervention while admitted to hospital following major thoracic surgery so we anticipate very few patients to crossover or be lost to follow-up. To reduce the risk of missing data, we will capture data from patient’s health records, surveys, and manual chart review. Any departures from the protocol will be documented in the participant file, reviewed, and compared quantitatively. Reasons for missing data will be recorded, and if missing data is >5%, multiple imputation methods will be used to address missing data in the analysis.

The statistical analysis will be performed using statistical software program R version 3.5.3. Continuous data that are normally distributed will be analyzed with an unpaired Student’s t-test. Non-continuous data will be analyzed with a chi-squared or Fisher’s exact test as appropriate. Level of significance will be determined by *p* < 0.05.

We plan to use this as a vanguard study. If a full-scale trial is completed, we plan to incorporate the results of this study into the data collection of a full-scale trial.

#### Interim analyses {21b}

The Data Safety Monitoring Board (DSMB) will meet either in-person or remotely to discuss matters related to the safety of study participants, validity and integrity of the data, enrollment rate relative to expectations, characteristics of participants, retention of participants, adherence to the protocol (potential or real protocol deviations), and data completeness.

The DSMB will review the interim data from the study once a total of 20 subjects have been treated with either the study drug or standard-of-care for at least 3 months, and again when all subjects have completed their 6-month study treatment. The DSMB may review the safety data at other times as warranted by emerging results. Based on review of the safety data, the DSMB can recommend continuation of the study without modification(s), study interruption, study termination, or modification of the trial, where applicable.

#### Methods for additional analyses (e.g., subgroup analyses) {20b}

Additional subgroup analysis by procedure type will be conducted.

#### Methods in analysis to handle protocol non-adherence and any statistical methods to handle missing data {20c}

The primary end point of this study is feasibility so no formal statistical analysis is planned. However, for the anticipated full-scale trial to follow, we plan to conduct analysis using an intention-to-treat, considering all patients as randomized regardless of whether they received the randomized treatment. Patients will be administered the intervention while admitted to hospital following major thoracic surgery so we anticipate very few patients to crossover or be lost to follow-up. To reduce the risk of missing data, we will capture data from patient health records, surveys, and manual chart review. Any departures from the protocol will be documented in the participant file and reviewed quantitatively to compare reasons. Reasons for missing data will be recorded, and multiple imputation methods will be used to address missing data in the analysis.

#### Plans to give access to the full protocol, participant-level data, and statistical code {31c}

There are no plans for granting public access to the full protocol, participant-level dataset, and statistical code beyond publication of this protocol.

### Oversight and monitoring

#### Composition of the coordinating center and trial steering committee {5d}

The research team includes three co-principal investigators: Andrew Seely, a thoracic surgeon and intensivist at The Ottawa Hospital, with experience leading clinical trials, will co-lead all phases of the study including recruitment, assessment, and analysis; Heather Smith, a General Surgery Resident in the Clinician Investigator Program at University of Ottawa, who led the validation of the predictive model, will co-lead the study including the assessment and analysis; and Salmaan Kanji, Pharm.D*.*, clinician scientist, with experience with AF trials, will contribute his expertise in drug safety/efficacy and oversee methods for dosing, preparation, and dispensing and will assist in methods and analysis. Co-investigators will contribute their expertise in clinical epidemiology and biostatistics, POAF, health technology assessment, and thoracic surgery.

The Ottawa Hospital Research Institute Internal Monitor will conduct a monitoring visit early on in the trial. This monitoring does not replace the routine quality control to be performed by the Principal Investigator or his designee.

#### Composition of the data monitoring committee, its role, and reporting structure {21a}

External oversight for this trial will be provided by an independent Data Safety and Monitoring Board (DSMB), consisting of one pharmacist and three clinician scientists. The DSMB will review matters related to the safety of study participants, validity and integrity of the data, enrollment rate relative to expectations, characteristics of participants, retention of participants, adherence to the protocol (potential or real protocol deviations), and data completeness.

#### Adverse event reporting and harms {22}

Amiodarone’s pharmacokinetic properties (namely its long half-life and prolonged washout period) as well the severe nature of some of its side effects make close monitoring essential among patients treated with this drug. Fortunately, many of the known adverse effects are secondary to tissue accumulation of amiodarone and are not seen with short-term therapy.

Below is a summary of how adverse events will be managed:

- *Allergic reaction*: If there is appearance of an allergic reaction, the study drug will be stopped immediately. Symptoms of the allergic reaction will be managed with supportive measures including antihistamines and intra-muscular epinephrine, as appropriate.
- *Hypotension*: If hypotension occurs with reasonable clinical suspicion that it is secondary to the intervention, treatment will be withdrawn. Hypotension will be treated with fluid resuscitation and vasopressor therapy, as indicated.
- *Bradycardia/AV blockade*: If bradycardia or AV blockade occurs with reasonable clinical suspicion that it is secondary to the intervention, treatment will be withdrawn. Severe bradycardia may require pharmacological treatment with atropine, dopamine, or epinephrine, or may require transcutaneous pacing. Our cardiology service will be consulted to assist with management as necessary.
- *ARDS*: If ARDS occurs (as defined under the Berlin definition), treatment will be withdrawn. ARDS will be managed according to standard of care with supportive treatments. Our respirology and/or intensive care teams will be consulted as needed to assist with management.
- *QT prolongation:* If QTc interval is prolonged with reasonable clinical suspicion that it is secondary to the intervention, treatment will be withdrawn. Any electrolyte abnormalities that would predispose the patient to cardiac arrhythmia will be corrected. Development of any ventricular arrhythmias will be managed in accordance with standard of care. Our cardiology service will be consulted to assist with management as necessary.

In the event of development of a significant adverse event, clinical follow-up will be arranged as necessary with our cardiology service.

Any events that occur after the patient starts the study intervention will be reported until postoperative day 30. All adverse events will be collected using the Common Terminology Criteria for Adverse Events (version 5.0) and reported on the applicable case report form [24]. All events will also be entered in an adverse event tracking log. Adverse events which are serious and unexpected, related or possibly related to the intervention, will be reported to the REB according to local requirements. Serious AEs (SAE) will be reported to Health Canada. If an event is determined to be serious, and related or possibly related to the intervention, regardless of expectedness, the Sponsor Investigator will notify Health Canada as soon as possible but no later than 7 calendar days after becoming aware of the event for fatal or life-threatening events, and no later than 15 days after becoming aware for non-fatal/life threatening events. Medical and scientific judgment will be exercised in deciding whether expedited reporting is appropriate in other situations.

#### Frequency and plans for auditing trial conduct {23}

The Ottawa Hospital Research Institute Internal Monitor will conduct a monitoring visit early on in the trial.

#### Plans for communicating important protocol amendments to relevant parties (e.g., trial participants, ethical committees) {25}

All protocol amendments will be reviewed and approved by the REB for prior approval or notification. The Principle Investigator will sign and date the approved protocol amendment prior to implementation. Any departures from the protocol will be documented in the participant file.

### Dissemination plans {31a}

The trial results will be disseminated to the public and to relevant clinical and academic communities. This will be done through submission for publication in an academic journal, through conference presentations, and to thoracic surgery patients via outreach to patient advocate groups and societies.

## Discussion

This trial is the first step to determining the ability for amiodarone to safely prevent POAF, specifically in high-risk patients after thoracic surgery. Preventing this adverse event will not only improve outcomes for patients but also reduce the associated health resource utilization and costs. This study leverages the previous work led by the Sponsor Investigator (AS) with the Canadian Association of Thoracic Surgeons (CATS) focused on capturing adverse events after thoracic surgery in a national database using standardized language across the country [3]. This study is the initial step to developing an RCT using the standardized classification to capture and grade POAF across multiple CATS-associated institutions to determine the effectiveness of amiodarone in preventing a complication.

This study is also the first to trial the concept of individualized prophylactic therapy targeted to high-risk individuals. We can identify individuals with a 4-fold increase of POAF who account for 51% of POAF cases using three simple factors available preoperatively [17]. Giving prophylactic amiodarone to only those high-risk individuals demonstrates an opportunity to optimize the benefits of prophylactic amiodarone and avoid unnecessary harm in individuals with low probability of developing POAF. We are increasingly aware that adverse events are more frequent in particular individuals, and in many cases, these patients can be identified preoperatively for interventions to prevent those complications. This study may serve as a template for future studies focusing on individualized prophylactic therapies to prevent adverse events captured by the CATS database.

## Trial status

This manuscript reflects the protocol version 4. The funding body has no role in the design of the study; in the collection, analysis, and interpretation of data; and in writing the manuscript dated May 19, 2020. Recruitment is anticipated for June 2021 and anticipated to be complete June 2022.

## Data Availability

Only the investigator team will have access to the final trial dataset.

## Abbreviations

AE: Adverse events
ARDS: Adult respiratory distress syndrome
DMP: Data Management Plan
DSMB: Data Safety Management Board
POAF: Postoperative atrial fibrillation
REB: Research Ethics Board
SAE: Serious adverse events
TM&M: Thoracic Morbidity And Mortality
